# Evaluation of the review models and approval timelines of authorities participating in the East African Medicine Regulatory Harmonisation initiative: alignment and strategies for moving forward

**DOI:** 10.3389/fmed.2024.1438041

**Published:** 2024-09-17

**Authors:** Nancy Ngum, Margareth Ndomondo-Sigonda, Rémy Habonimana, Fred Siyoi, Clarisse Irasabwa, Julia Ojukwu, Felchism Apolinary, Andrew Okello, Sabrina Ahmada, Stuart Walker, Sam Salek

**Affiliations:** ^1^Department of Clinical and Pharmaceutical Sciences, School of Life and Medical Sciences, University of Hertfordshire, Hatfield, United Kingdom; ^2^African Union Development Agency—New Partnership for Africa’s Development, Johannesburg, South Africa; ^3^Burundi Food and Medicines Regulatory Authority, Bujumbura, Burundi; ^4^Pharmacy and Poisons Board, Nairobi, Kenya; ^5^Rwanda Food and Drugs Authority, Kigali, Rwanda; ^6^Drug and Food Control Authority, Juba, South Sudan; ^7^The Tanzania Medical Devices Authority, Dodoma, Tanzania; ^8^National Drug Authority, Kampala, Uganda; ^9^Zanzibar Food and Drugs Authority, Zanzibar, Tanzania; ^10^Centre for Innovation in Regulatory Science, London, United Kingdom; ^11^Institute of Medicines Development, London, United Kingdom

**Keywords:** East African Medicines Regulatory Harmonisation (EAC-MRH), joint assessment procedure, regulatory review models, regulatory reliance, African Medicines Agency (AMA)

## Abstract

**Introduction:**

Medicines regulatory harmonisation has been embraced by many national regulatory authorities (NRAs) to improve public health through faster availability of safe, high-quality, and effective medical products to patients and enhanced standardisation of technical guidelines and work sharing, leading to reduced cost to pharmaceutical companies. After ten years of implementing regulatory harmonisation by the East African Community Medicines Registration Harmonization (EAC-MRH) initiative, it is now imperative for participating NRAs to rely on each other to minimise duplication of use of limited resources. Major challenges in implementing reliance are the lack of clear registration processes and delays in the approval. The aim of this study was to compare review models, target timelines and data requirements used in assessing applications by EAC-MRH NRAs so as to align and propose strategies for improvement.

**Methods:**

A validated questionnaire that standardises and captures review processes was completed by the head of the medicine’s registration division in each of the seven EAC-MRH NRAs. A country report based on the completed questionnaire was developed for each NRA and validated by the heads of the respective authorities.

**Results:**

Most applications received by all countries were for generics except Kenya, which received a significant number of new active substance applications (55 and 53 in 2020 and 2021). Mean approval times for generics using full review varied, with Tanzania’s time declining for the three years. Target timelines for full review for the five countries ranged between 180 calendar days (Tanzania) to the highest 330 days (Zanzibar). The three countries (Kenya, Rwanda and Uganda) utilising the verification review model had a target timeline of 90 days. All six authorities conducted abridged reviews and fast-track assessments through a priority review track. The common technical document format was mandatory for applications in all authorities. The target timeline for key milestones in the review process varied for each country with a few similarities.

**Discussion:**

The study has provided a baseline for review models, target timelines and data requirements utilised in assessing applications for registration by EAC-MRH NRAs. Implementing the recommendations from this study will enable the NRAs to align and improve their registration processes.

## 1 Introduction

One of the key functions of national medicines regulatory authorities (NRAs) is the review of applications and registration of medical products submitted by pharmaceutical manufacturing companies. NRAs are expected to have effective and efficient regulatory systems to ensure that timely marketing authorisation is granted for safe, effective and good-quality medical products. One of the objectives of establishing the East African Community Medicines Registration Harmonization (EAC-MRH) project was to build the capacity of NRAs in the region through work sharing, training, and twinning. Currently there is a strong advocacy for reliance, especially as most of these authorities delay issuing marketing authorisation for medical products, leading to a significant backlog.

Over several years, the process of medicines regulatory harmonisation has been embraced by many NRAs to improve public health through faster availability of safe, high-quality, and effective medical products to patients. This has enhanced the harmonisation of technical guidelines and work sharing, leading to reduced costs to pharmaceutical companies as they prepare one single set of applications to submit to several countries. After ten years of implementing regulatory harmonisation by the EAC NRAs, it is now imperative for these NRAs to rely on each other so as to minimise duplication of their use of limited resources. One of the major challenges in implementing reliance; however, is the lack of clear registration processes in the NRAs and the delay in the approval of medical products.

### 1.1 Reliance

With the complexities that come with the granting of marketing authorisation for medical products, most regulatory authorities are now embracing the concept of reliance as a way of improving performance. It is now clear that no one authority can do it all, especially with new advanced health technologies and emerging diseases plaguing the world. The main objectives of harmonisation initiatives are to build trust amongst NRAs so that they can rely on each other’s decisions. According to the World Health Organisation (WHO) guidelines on good reliance practices, NRAs are encouraged to implement reliance to minimise duplication of effort especially given their limited resources. Countries with weak regulatory systems are called upon to rely on WHO-listed authorities (WLAs). According to the (1) R&D Briefing 93, in the past five years there has been an increase in the use of facilitated regulatory pathways for approval of new medicines, even by well-resourced NRAs but regulatory reliance and work sharing will especially help low- and middle-income countries to have access to innovative medicines in a timely manner (2).

### 1.2 Registering medical products in low-to-middle income countries

The main function of NRAs is to register medical products in their countries. This is also known as granting marketing authorisation or product licensing (3). Countries have different regulatory requirements for the registration of pharmaceutical products. Understanding the review models and approval timelines for the East African Community as an emerging market for pharmaceutical companies is critical (4) in fast tracking the registration process to provide the much-needed medical products to patients in a timely manner. There has been a general indication that for applicants interested in these markets, NRAs should ensure that the application procedures are clear, that communication and transparency is enhanced, with timelines for approval of products clearly outlined, and with registration guidelines for countries in the same region being harmonised and registration processes being effective and efficient (5, 6).

However, reviewers have also raised the challenge that long review timelines experienced in the registration of medical products are sometimes caused by the delay in manufacturers’ or applicants’ response to queries. It is therefore important to understand that regulatory authority requirements for review models should inform the industry and other stakeholders what to expect from the authorities.

The first paper of this series focused on comparing the key milestones in the review process using a general model with a process map and milestones. It also examined how these authorities build quality into the review by analysing their good review practices and how quality is built into the decision-making practices of the EAC NRAs and whether there are measures in place to guide good decisions.

The aim of this paper, which is the second of this series is to compare the review models, target timelines and data requirements utilised in assessing applications for registration by countries participating in the EAC-MRH initiative so as to align and propose strategies for improvement.

## 2 Materials and methods

### 2.1 Study participants

The study participants included Senior Programme Officers from the Medicines registration divisions in the seven NRAs; Pharmacy and Poisons Board-PPB, Kenya; National Drug Authority-NDA, Uganda; The Tanzania Medical Devices Authority (TMDA); Zanzibar Food and Drugs Authority (ZFDA) Tanzania; Drug and Food Control Authority DFCA South Sudan; Burundi Food and Medicines Regulatory Authority (ABREMA) and Rwanda Food and Drugs Authority.

According to rules of the Ethics Committee of the University of Hertfordshire, as the study participants were not patients or healthcare professionals working in healthcare facilities, the researcher was permitted to use informed implied consent; that is, by agreeing to participate in the study and complete the questionnaire, the participants had implicitly provided their consent.

### 2.2 Data collection

A validated questionnaire (Optimising Efficiencies in Regulatory Authorities: OpERA) describing the organisation structures, regulatory review systems for market authorisation of new active substances (NASs) and generics, including their overall timelines from the date of submission of the application to when it is approved, good review practices (GrevP) and quality decision-making practices, was completed by each of the authorities in 2022 and 2023. The questionnaire is composed of six different parts: Part 1 documents the organisation of the authority with the focus on its structure and resources; Part 2 covers the types of review models used by the authority for the scientific assessment of medicines; Part 3 is based on key milestones in the review process with the focus on the process map and milestones; Part 4 relates to good review practices (GrevP) and how an authority builds quality into their regulatory processes; Part 5 focuses on the quality of the decision-making processes based on whether the authority have good measures in place to guide decision making; and Part 6 describes the challenges and opportunities available to the national regulatory authorities.

### 2.3 Models of regulatory review

A risk-based approach to review involves different review models that describe the ways in which authorities assess the scientific data received from applicants during the assessment process. This can vary depending on whether the data are assessed in detail by the authority, or the authority relies on results of the assessment conducted elsewhere. The decision to choose a type of review model will also depend on the type of product and its status with other authorities.

The different steps in the review process do have a significant effect on the review timelines and subsequent market authorisation. There are three types of review models that NRAs can use:

The verification review (type 1) is used to minimise duplication by allowing a product that has been registered in a recognised authority to be marketed in the receiving country. The main responsibility of the receiving country is to verify that the product has indeed been registered elsewhere and is exactly the same product.

The abridged review (type 2) model also minimises the use of resources by not reviewing scientific data that have been assessed elsewhere but focusing on reviewing the product based on its local conditions, which could be climate, infrastructure for distribution, benefit-risk assessment, and medical practice culture.

The full review (type 3) is employed when the authority assesses the complete application including all the scientific data. This is carried out with applications that have not been reviewed elsewhere and requires more human resources and an improved infrastructure.

## 3 Results

For the purpose of clarity, the results of this study will be presented in three parts: Part 1: Metrics of applications received and registered; Part 2: Review models, extent of scientific assessment and data requirements and Part 3: Targets of key milestones in the review process.

### 3.1 Part 1: Metrics on NASs, generics, and WHO prequalified generics

All seven countries completed the OpERA Questionnaire. However, South Sudan did not report any data since they had not received any applications for the specified study period. Kenya received 55 applications for NASs in 2020 and approved 18 and received 53 applications in 2021 out of which 47 were approved. In 2022 Rwanda received 409 applications for NASs and approved 160 and in 2023 received 398 applications and approved 60 (Table 1).

**TABLE 1:** Comparison of metrics for NASs, generics, and WHO-prequalified generics (2020–2023).

Coun-try	Burundi				Kenya				Rwanda				Tanzania				Uganda				Zanzibar			
**Year**	**2020**	**2021**	**2022**	**2023**	**2020**	**2021**	**2022**	**2023**	**2020**	**2021**	**2022**	**2023**	**2020**	**2021**	**2022**	**2023**	**2020**	**2021**	**2022**	**2023**	**2020**	**2021**	**2022**	**2023**
**NASs**																								
Received	0	0	0	0	55	53	N/S	N/S	0	0	409	398	0	0	0	0	NS	NS	0	0	0	0	0	0
Approved	0	0	0	0	18	47	N/S	N/S	0	0	160	60	0	0	0	0	NS	NS	0	0	0	0	0	0
**Generics**																								
Received	157	68	80	342	692	909	N/S	N/S	533	615	390	379	631	975	1,079	764	508	849	804	905	8	10	14	22
Approved	110	0	36	57	81	368	N/S	N/S	46	55	147	51	499	383	359	51	389	405	430	571	1	2	0	0
**WHO pre-qualification**																								
Received	0	2	0	1	10	35	N/S	N/S	16	18	7	3	7	22	16	14	10	12	7	6	1	0	0	0
Approved	0	0	4	1	10	20	N/S	N/S	0	11	7	0	7	14	13	12	10	12	7	3	1	0	0	0

NASs, new active substances; WHO, World Health Organization; N/S, not specified.

**FIGURE 1:**
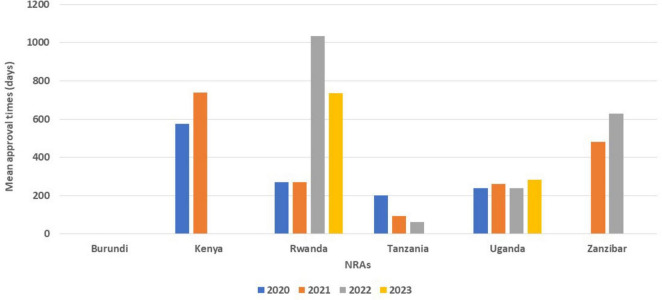
Comparison of number of generics approved from 2020 to 2023.

All the six NRAs received applications for generics, with Tanzania approving the highest number of applications (499) for 2020 and (503) 2021. It is interesting to note that the number of generics approved by Tanzania dropped in 2022 to 359. Kenya received more applications (692) in the same year (2020), but only granted marketing authorisation for 81 products. Burundi in 2020 received 157 applications and approved 110 but in 2023 approved 57 with 342 applications received. In 2021, Kenya received 909 applications and only approved 368 while Uganda received 849 and approved 405. Burundi on the other hand did not approve any product in 2021 even though they received 68 applications. Uganda received the highest number (849) of applications in the region in 2021 and was able to register 405 generic products during the year. Tanzania in 2021 received 704 applications and registered 503 while Zanzibar received 10 applications in the same year but only approved two in 2022 (Figure 1).

Kenya and Rwanda saw a slight increase in WHO pre-qualified generics approved in 2021 while Burundi and Zanzibar did not receive WHO pre-qualified applications. Tanzania in 2021 received 15 WHO pre-qualified applications and approved 13. For Uganda there has been a decline in the number of WHO pre-qualified applications from 2021 to 2023 (Table 1).

### 3.2 Mean approval times

**FIGURE 2:**
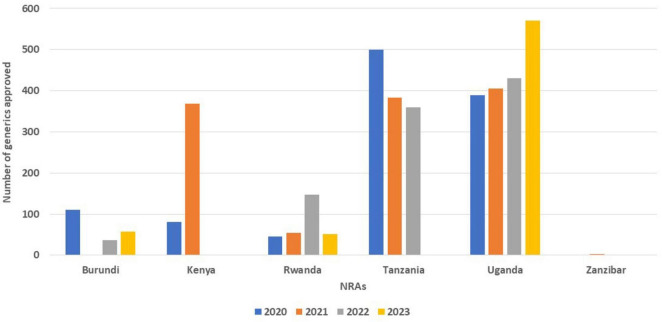
Comparison of mean approval times for generics using full review from 2020 to 2023.

While Kenya received a number of applications for NASs, they approved 18 applications in 2020 and 47 applications in 2021 (Table 1), but they did not indicate the mean approval times for a full review of NAS applications (Table 2). Tanzania saw a decline in the mean approval times for the full review of generics in three consecutive years (202 days in 2020, 93 days in 2021 and 61 days in 2022). Rwanda took 1,035 days for the full review of generics in 2022, which declined to 735 days in 2023, while full review of generics in Kenya increased from 575 days in 2020 to 739 days in 2021. The mean approval timelines for generics in Uganda saw a slight decrease in 2022 (238 days) from 261 days in 2021; however, there was an increase in 2023 to 284 days (Figure 2).

**TABLE 2:** Comparison of mean approval times NASs, generics and WHO prequalified generics 2020–2023 (calendar days).

Coun-try	Burundi				Kenya				Rwanda				Tanzania				Uganda				Zanzibar			
**Year**	**2020**	**2021**	**2022**	**2023**	**2020**	**2021**	**2022**	**2023**	**2020**	**2021**	**2022**	**2023**	**2020**	**2021**	**2022**	**2023**	**2020**	**2021**	**2022**	**2023**	**2020**	**2021**	**2022**	**2023**
**Full review**																								
NASs	N/A	N/A	N/A	N/A	N/V	N/V	N/V	N/V	N/A	N/A	N/A	N/A	N/A	N/A	N/A	N/A	0	0	N/A	N/A	0	0	0	
Generics	N/A	N/A	N/A	N/A	575	739	N/V	N/V	270	270	1,035	735	202	93	61	85	237	261	238	284	0	480	630	
WHO Pre-qualifi cation	N/A	N/A	90	90	N/A	341	N/V	N/V	90	90	484	90	83	N/A	N/A	79	54	60	56	65	0	0	0	
**Verification**																								
NASs	N/A	N/A	N/A	N/A	N/V	N/V	N/V	N/V	N/V	N/V	N/V	N/V	N/A	N/A	N/A	N/A	N/A	N/A	N/A	N/A	N/A	N/A	N/A	N/A
Generics	N/A	N/A	N/A	N/A	N/V	N/V	N/V	N/V	N/V	N/V	N/V	N/V	N/A	N/A	N/A	N/A	N/V	N/V	54	43	0	0	78	0
WHO Pre-qualifi cation	N/A	N/A	90	90	N/V	N/V	N/V	N/V	N/V	N/V	N/V	N/V	N/A	N/A	N/A	N/A	54	60	56	65	90	90	90	
**Abridged**																								
NASs	N/A	N/A	N/A	N/A	N/V	N/V	N/V	N/V	N/V	N/V	N/V	N/V	N/A	N/A	N/A	N/A	N/A	N/A	N/A	N/A	0	0	0	
Generics	N/A	N/A	N/A	N/A	N/V	N/V	N/V	N/V	N/V	N/V	N/V	N/V	241	153	93	N/A	N/V	N/V	N/V	N/V	180	0	0	
WHO Pre-qualifi cation	N/A	N/A	90	90	N/V	N/V	N/V	N/V	N/V	N/V	484	90	N/A	14	13	N/A	N/V	N/V	N/V	N/V	0	0	0	

N/A, not applicable; N/V, not available.

For WHO pre-qualified applications, Rwanda (484 days) and Kenya (341 days) took a longer mean approval times using full review while the other countries took less than 100 days for the approval of generics (Table 2).

Using verification review, authorities in Burundi and Zanzibar took an average of 90 days in 2022 to review WHO pre-qualification applications. Zanzibar also reported taking a mean approval time of 78 days to review EAC-MRH-recommended applications. From 2020 to 2023, Uganda reported mean approval times of less than 65 days for generics and WHO pre-qualified products. Kenya and Rwanda did not report the mean approval times for verification review type for NASs, Generics and WHO pre-qualified applications (Table 2).

For the abridged review type, Zanzibar spent 180 days in 2020 as mean approval times for generics. Burundi took 90 days in 2022 for WHO pre-qualification while Tanzania took 14 days in 2021 and 13 days in 2022. In 2021, Rwanda took 484 days for approval of WHO pre-qualification application. Kenya and Rwanda did not submit information on mean approval times when using the abridged review and verification types (Table 2).

### 3.3 Part II: Review models used for scientific assessment

All of the six authorities carry out full and abridged reviews for scientific assessment.

#### 3.3.1 Verification review (type 1)

Burundi, Tanzania and Zanzibar do not conduct verification reviews for generics. However, Burundi and Zanzibar do use verification review for WHO-prequalified and EAC-MRH- recommended applications. The reason for not implementing type 1 assessment by TMDA is that they do not employ mutual recognition policies yet. The authority offers special import permits based on its regulations. Kenya and Rwanda conduct verification reviews for selected applications like WHO pre-qualified and WLA-approved products, and authorities who have valid agreements to share reports. For Uganda, verification reviews are used for WHO collaborative registration procedures (CRP) and EAC-recommended products (Table 3).

**TABLE 3:** Review models employed and target timelines (calendar days—2022–2023).

Type of review model	Burundi	Kenya	Rwanda	Tanzania	Uganda	Zanzibar
Verifications review (type 1)	x	✓c	✓c	x	✓a	x
Target	N/A	90	90	N/A	90	N/A
Abridged review (type 2)	✓b	✓c	✓c	✓c	✓e	✓c
Target	N/A	105	90	126	105	126
Full review (type 3)	✓3A	✓3B	✓3A	✓3B	✓3A	✓3A
Target	N/A	262	270	180	261	365
Fast track/priority review	✓	✓	✓	✓	✓	✓
Target	N/A	N/A	N/A	90	N/A	126

a: For World Health Organization (WHO) collaborative registration procedure (CRP) and East African Community (EAC)-recommended products. b: For WHO CRP, WHO-listed authority (WLA)-approved and EAC-recommended products. c: For WHO-prequalified and WLA-approved products. e: For OTC products.

Reference authorities used by the NRAs include WHO-prequalification programme authorities, International Council for Harmonisation of Technical Requirements for Pharmaceuticals for Human Use (ICH) founding members and WLAs such as Swissmedic, European Medicines agency (EMA), United States Food and Drug Authority (US FDA), South Korea, Singapore and EU Medicines Network. In addition to WLAs listed above, East African Community work sharing Initiative (EAC-MRH), Intergovernmental Authority on Development (IGAD), TMDA and Ghana FDA were also reference authorities for PPB. All three countries had a 90-day target time for the verification review.

#### 3.3.2 Abridged review (type 2)

All six authorities conducted abridged reviews. Type 2 assessment is used by Burundi-ABREMA for selected applications such as products that have been registered by WHO, WLAs, PPB, NDA, TMDA and EAC-recommended products. While Kenya, Rwanda, Tanzania, and Zanzibar use abridged reviews for selected applications that were previously approved by WHO-prequalified and WLA-approved products. For Tanzania, these selected applications must be approved in at least two reference countries, and not rejected in any other reference country. Uganda utilises the abridged review pathway for over-the-counter (OTC) products. Products category reviewed by Zanzibar are NAS, major line extensions, generics and biosimilars. Kenya and Uganda have a target time of 105 calendar days, Rwanda 90 calendar days, and Tanzania 126 days (Table 3).

#### 3.3.3 Full review (type 3)

All six authorities conduct type 3 assessment for all applications that do not qualify for type 1 or type 2 data assessments. Only Kenya and Tanzania conduct Type 3B [a full, independent review of pre-clinical (safety) and clinical (efficacy) is carried out] for all major applications. The other authorities conduct type 3A in which data on quality, pre-clinical (safety) and clinical (efficacy) are assessed in detail but there are requirements for pre-registration elsewhere before the authorisation can be finalised (Table 3).

Only Burundi did not have a target time for full review of applications. Tanzania had the lowest target time for full review of 180 calendar days, followed by Uganda, 261 days, Kenya, 262 days, Rwanda, 270 days, and Zanzibar, 365 days (Table 3). Table 6 provides additional data for these targets with respect to major milestones.

#### 3.3.4 Fast-track/priority review

All six authorities conduct fast-track assessments through a priority review system. Only Tanzania and Zanzibar indicated a target timeline of 90 and 126 calendar days, respectively, for review of fast-tracked applications in 2022 (Table 3). The authorities conduct a rapid assessment of the application to obtain pharmacological, marketing/commercialisation, pharmacovigilance, and additional clinical trials information. Applicants were charged a higher fee for priority review that achieve a shorter timeline.

#### 3.3.5 Data requirements

In all six authorities a Certificate of a Pharmaceutical Product (CPP) is required to be submitted with an application or before authorisation is issued. A common technical document (CTD) format is mandatory for applications in all authorities and all review types, require submission of full data for Modules 1–5 and summary data for modules 2.3, 2.4 and 2.5 (Table 4).

**TABLE 4:** Summary comparison of key features of the regulatory systems for medicines.

Marketing authorisations	Burundi	Kenya	Rwanda	Tanzania	Uganda	Zanzibar
Certificate of Pharmaceutical Product (CPP) is required with the application or before authorisation is issued	✓	✓	✓	✓	✓	✓
Common technical document (CTD) format is mandatory for applications	✓	✓	✓	✓	✓	✓
Medical staff: More than 25% within the authority review staff are physicians	x	x	x	x	x	x
Review times: The authority sets targets for the time it spends on the scientific assessment of NASs and generic applications	✓	x	✓	✓	✓	x
Approval times: The has a target for the overall time for the review and approval of an application	✓	✓	✓	✓	x	✓
Questions to sponsors are batched at fixed points in the review procedure	✓	✓	✓	✓	✓	✓
Company response time: Recording procedures allow the company response time to be measured and differentiated in the overall processing time	✓	✓	✓	✓	x	✓
Priority reviews: The authority recognises medical urgency as a criterion for accelerating the review and approval process for qualifying products	✓	✓	✓	✓	✓	✓
Sequential processing: Different sections of technical data reviewed sequentially rather than in parallel	x	x	x	✓	x	x
Price negotiation: Discussion of pricing is separate from the technical review and does not delay the approval of products	x	✓	x	x	✓	✓
Sample analysis: The focus is on checking quality in the marketplace and requirements for analytical work do not delay the marketing authorization	✓	x	x	✓	✓	✓

The authorities then conduct a detailed assessment, and prepare an evaluation report. Factors considered in assessing product risks and benefits include differences in medical culture/practice, ethnic factors, and national disease patterns. The authorities also endeavour to obtain internal assessment reports from other authorities such as the referenced authorities, public assessment reports available through the internet such as the European Public Assessment Reports (EPARs) or through participation in the WHO collaborative registration procedure where access is given to reports of prequalified products. All six authorities also have access to reports assessed through the EAC-MRH initiative, as part of participation in the EAC-MRH programme. A primary scientific review is conducted by the authority staff, although Tanzania also includes external reviewers.

Apart from Kenya and Zanzibar, the other four authorities set targets for review times spent on the scientific assessments. Only Uganda does not have a recording procedure that allows the company response time to be measured. All the authorities recognise medical urgencies and thus implement priority reviews for qualifying products. Only Tanzania conducts sequential processing of technical data. For all six authorities, physicians comprise less than 25% of the authority medical review staff. All the authorities have an approval times target for the overall time for the review and approval of an application (Table 5).

**TABLE 5:** Extent of scientific assessment for full review.

	Burundi	Kenya	Rwanda	Tanzania	Uganda	Zanzibar
Chemistry, manufacturing and control (CMC) data extensive assessment				✓		✓
Non-clinical data extensive assessment	✓	✓	✓	✓	✓	✓
Clinical data extensive assessment	✓	✓	✓	✓	✓	✓
Bioequivalence data extensive assessment				✓		
Additional information obtained (where appropriate)	✓	✓	✓	✓	✓	✓
Other agencies internal review reports	✓	✓	✓	✓	✓	✓
Medical and scientific literature	✓			✓		

### 3.4 Part III: Targets for key milestones in the review process

In line with good review practices, each regulatory authority should set a target timeline for each milestone and the overall process. In the first article of this series, the review process, and key milestones for the six authorities were reported. This article reviews the target timelines for these key milestones. A standardised process map for review and approval of medical products demonstrates key milestones that are usually recorded and monitored by mature regulatory authorities in the review of applications.

#### 3.4.1 Receipt and validation

Uganda had no target time for receipt and validation of applications. Kenya has the shortest target time of 3 days, followed by Tanzania with 5 calendar days, and Rwanda with 30 days. Both Burundi and Zanzibar have 90 calendar days as their target (Table 6).

**TABLE 6:** Comparison of targets for key milestones in the full (type 3) review process -(calendar days).

Target	Burundi	Kenya	Rwanda	Tanzania	Uganda	Zanzibar
Receipt and validation (A–B)	90	3	30	5	No target time	90
Queuing (B–C)	60–180	< 365	60–150	35	365	60–180
Primary scientific Assessment (C–D)	90	No target time	No target time	100	180	180
Questions to applicant (Clock stop) (D–E)	90	180	90	180	180	180
Review by Expert Committee (G–H)	90	No target time	60	1	30	1
Approval procedure (Admin)	30–90	< 30	< 30	< 30	30–90	< 30
Overall approval time (A–I)	90	730	365	180 (exc. Applicant time)	547	365

A for biosimilar products not approved by a reference authority only.

#### 3.4.2 Queue time

Queue time is that time taken to start the scientific assessment after the application has been validated or accepted for review. Uganda and Kenya have the longest queue time of 365 days, followed by Burundi, Rwanda and Zanzibar with queue times ranging from 60 to 180 calendar days. Tanzania had the shortest queueing time of 35 calendar days (Table 6).

#### 3.4.3 Primary scientific assessment

Burundi had the shortest target for primary scientific assessment of 90 calendar days followed by Tanzania with 100 days, including peer review. Uganda and Zanzibar have primary scientific assessment target times of 180 days. Kenya and Rwanda did not have target times (Table 6)

#### 3.4.4 Questions to applicants

Here the clock stops as the assessment is paused and time given to the sponsor to respond to any queries. The target for clock stops is 90 days for Burundi and Rwanda, and 180 days for Kenya, Tanzania, Uganda, and Zanzibar (Table 6).

#### 3.4.5 Review by expert committee

Four of the authorities use expert committees to make decisions on approval or refusal of marketing authorisation of medical products. Zanzibar does not use expert committees; Tanzania takes one day to make the expert committee decision while Uganda takes 30 days followed by Burundi with 90 days. Kenya does not have a target time (Table 6).

#### 3.4.6 Authorisation procedure

This is the time it takes to issue the overall approval after the scientific opinion has been made. Four of the authorities (Kenya, Rwanda, Tanzania, and Zanzibar) take less than 30 days. Uganda takes 30 to 90 days; however, the sponsor is informed of a positive scientific opinion before the authorisation is issued, whereas Burundi does not give a target (Table 6).

## 4 Discussion

The aim of this study was to compare the review models, target and review timelines as well as data requirements utilised in assessing applications for registration by countries participating in the EAC-MRH initiative to align and propose strategies for improvement. Countries with higher populations received higher numbers of applications and are also autonomous authorities. Ozawa et al. (7) demonstrate how improving the autonomy of health facilities improves access to essential medicines.

It is interesting to note that only one country in the region received applications for NASs in 2020 and 2021. This is not surprising, as several studies have highlighted that that the number of NASs launched in low- and middle-income countries are very few as compared to high-income countries (5, 8). Most innovative medicines or new medicines are usually first approved by well-resourced regulatory authorities (3). The study by Centre for Innovation in Regulatory Science [CIRS] (1) reported how six major regulatory authorities (Europe, USA, Japan, Canada, Switzerland and Australia) have used facilitated regulatory pathways and internationalisation for approvals of new medicines. It is hoped that many new and complex molecule applications will be submitted through the operationalisation of the African Medicines Authority (AMA).

It would be important to understand the reason for a decline in the number of applications received and approved by Burundi in 2021 as compared to 2020 and the decrease in mean approval times for generics in Tanzania from 202 days in 2020 to 61 days in 2022.

All six authorities in the region are implementing reliance, as the majority employ the verification and abridged review models (9, 10). It is important to note that countries in this region are already relying on each other, which is the major success of the EAC work-sharing initiative. To enhance collaboration, it will be critical for these countries to have mutual recognition or cooperation agreements especially for Tanzania, which is unable to implement the verification review due to the absence of mutual recognition agreements. It is also going to be beneficial for inter-regional economic community (REC) reliance to be instituted for the REC-MRH initiatives so that the different regions can also rely on the decisions of each other. This study provided a clear understanding of the review processes and regulatory requirements for registration of medical products in the authorities in East Africa. This will act as a baseline for future studies especially when there will be need to evaluate progress and identify any improvements as the AMA becomes operationalised. Other authorities have also been given the opportunity to better understand these review processes and can learn from each other as they share experiences.

### 4.1 Recommendations

As a result of this study, the following recommendation should be considered by the six authorities taking part.

1. EAC-MRH as a reference authority: All authorities participating in the EAC-MRH initiative should consider formally recognising EAC-MRH as a reference authority for a reliance pathway.
2. Timelines and targets: Authorities should consider documenting all key milestones and relevant timelines in order to monitor and measure their regulatory performance.
3. Information system: NRAs should develop information systems that can track registration timelines from the date the application is received to the date the registration is granted.
4. Mutual recognition: Develop and implement mutual recognition agreements to enhance reliance practices amongst NRAs in the region as well as inter-REC reliance.
5. Communication to applicants: All authorities should communicate their regulatory requirements to applicants on their website in order to facilitate a seamless review process as well as improve timelines.

6. Capacity building: Authorities should consider the following:

• Exchange of staff between authorities
• Secondments
• In-house education and training and continuous professional development

### 4.2 Study limitations

This study focuses on East Africa region and the respective national regulatory authorities; while it provides detailed insights into the EAC-MRH initiative, the findings may not be generalisable to other regions or global regulatory practices.

In addition, South Sudan did not report any data since they had not received any applications for the specified study period. Furthermore, Kenya and Rwanda did not record information on mean approval times for different review models.

Whilst this study provides a broad overview of the quantitative data obtained from the questionnaire, it lacks in-depth qualitative insight from the stakeholders that would have added more context to the findings.

Given the extent of the quantitative data collected by the Questionnaire, it would have been desirable to also collect qualitative data through interviews and focus groups involving regulatory officials, pharmaceutical companies, and healthcare professionals in order to provide richer context for the quantitative findings.

Although the limitations of the study have the potential of introducing biases to the findings, this is believed to be minimal since the design of the study was “hypothesis generating” as opposed to “hypothesis testing.” This means that factual aspects of the findings were reported without extensive extrapolation of the results.

## 5 Conclusion

This study serves as the first comparative evaluation of review models for the NRAs of the EAC countries. It has provided a baseline for review models, and target and review timelines as well as data requirements utilised in assessing applications of medical products for registration by countries participating in the EAC-MRH initiative. It is important for NRAs to have open-minded discussions, document best practices and share experiences so as to learn from each other or from reference authorities. Reliance mechanisms should be developed and implemented by the countries in the region. Implementing the recommendations from this study will enable the NRAs to align and improve their registration processes.

## Data availability statement

The raw data supporting the conclusions of this article will be made available by the authors, without undue reservation.

## Author contributions

NN: Conceptualization, Formal analysis, Writing – original draft, Writing – review & editing. MN-S: Writing – review & editing. RH: Data curation, Writing – review & editing. FS: Data curation, Writing – review & editing. CI: Data curation, Writing – review & editing. JO: Data curation, Writing – review & editing. FA: Data curation, Writing – review & editing. AO: Data curation, Writing – review & editing. SA: Data curation, Writing – review & editing. SW: Conceptualization, Formal analysis, Writing – original draft, Writing – review & editing. SS: Conceptualization, Formal analysis, Writing – original draft, Writing – review & editing.
